# Supplementary material for: Pick’s Tau Fibril Shows Multiple Distinct PET Probe Binding Sites: Insights from Computational Modelling
Source: Int J Mol Sci. 2020 Dec 31;22(1):349. doi: 10.3390/ijms22010349 (PMC7796283; doi:10.3390/ijms22010349)
Supplement: Supplementary file 1 [file ijms-22-00349-s001.zip › Supplementary files/Supplementary Figures.pdf]

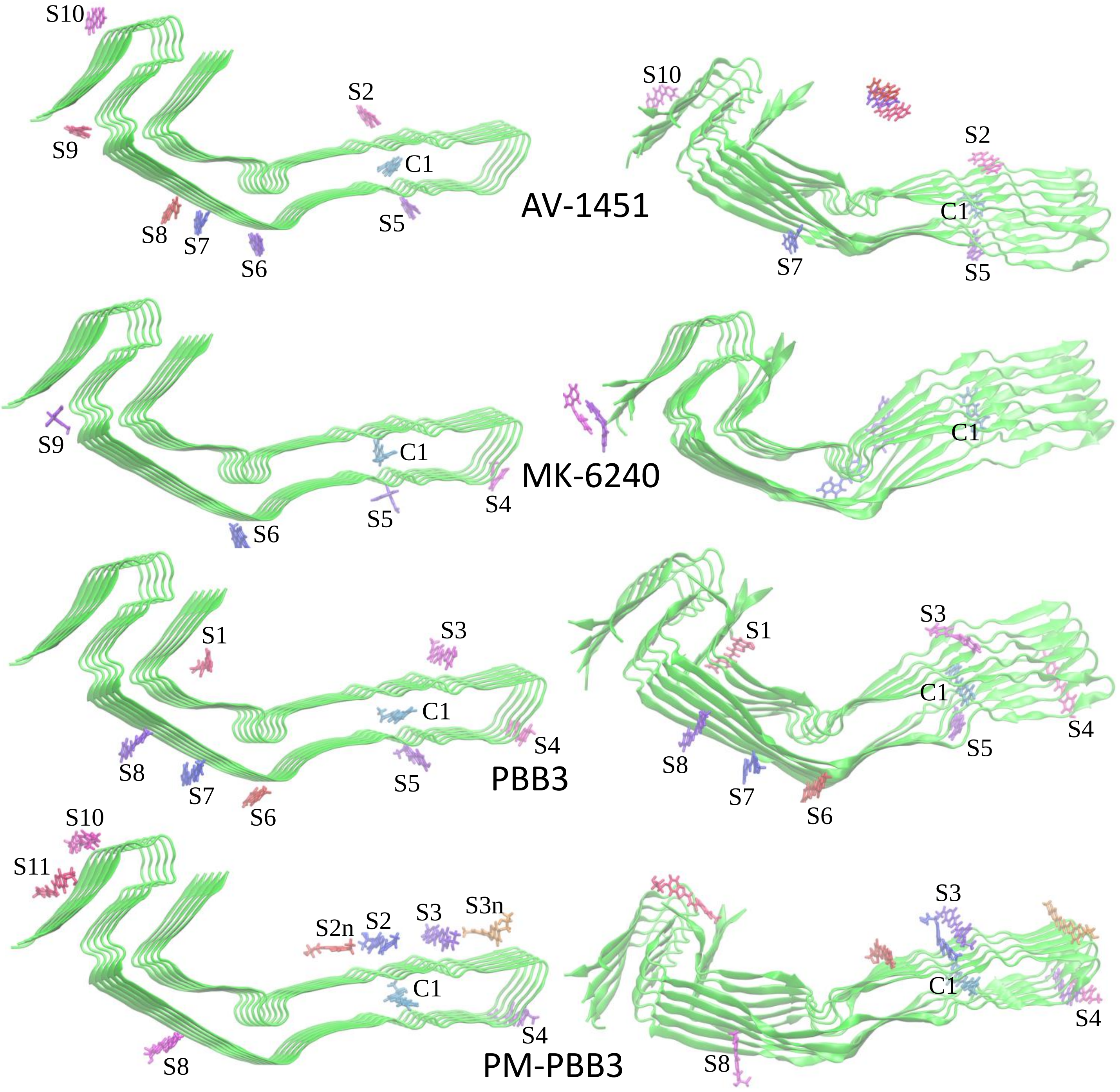

Supplementary Figure S1 Initial PET probe-bound tau structures (left) and the structures after MD simulation (right). In the initial structures, all the possible binding sites are docked with PET probes, AV-1451, MK-6240, PBB3 and PM-PBB3.

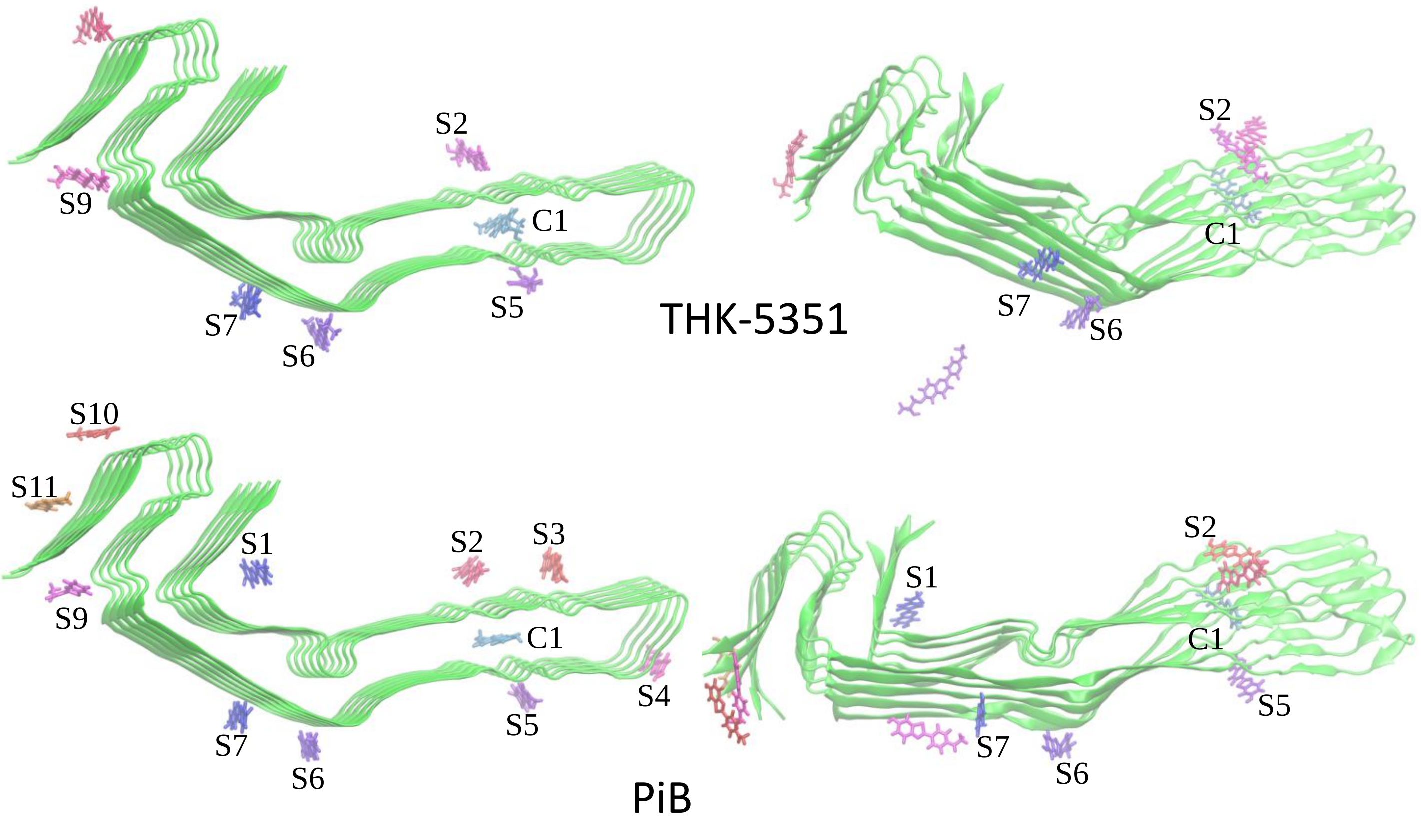

Supplementary Figure S2 Initial PET probe-bound tau structures (left) and the structures after MD simulation (right). In the initial structures, all the possible binding sites are docked with PET probes, THK-5351 and PiB.

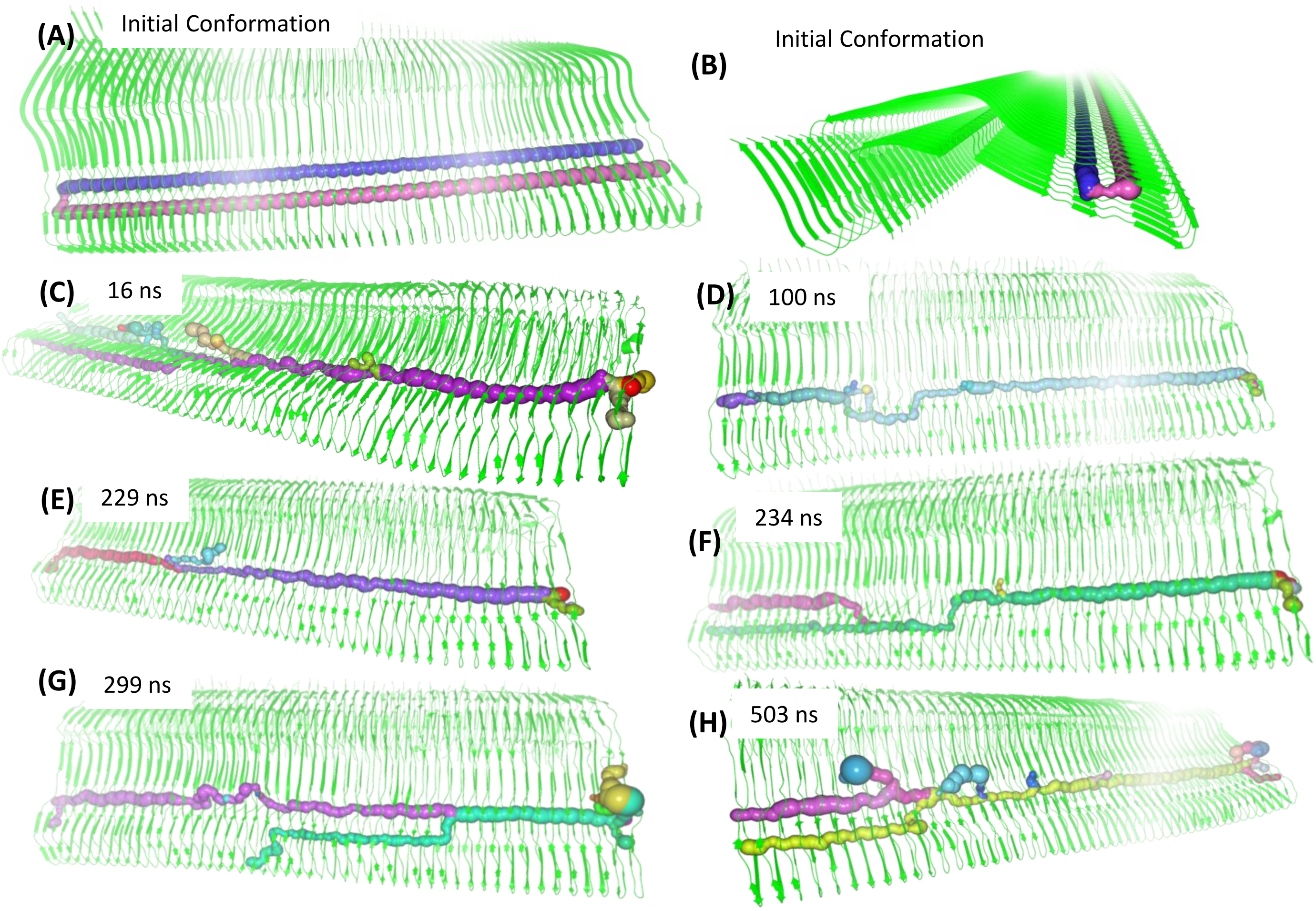

Supplementary Figure S3 Tunnels calculated in the tau cryo-EM structure (A, B) and structure from MD simulation of the after (C) 16 ns, (D) 100 ns, (E) 229 ns, (F) 234 ns, (G) 299 ns, and (H) 503 nanoseconds.

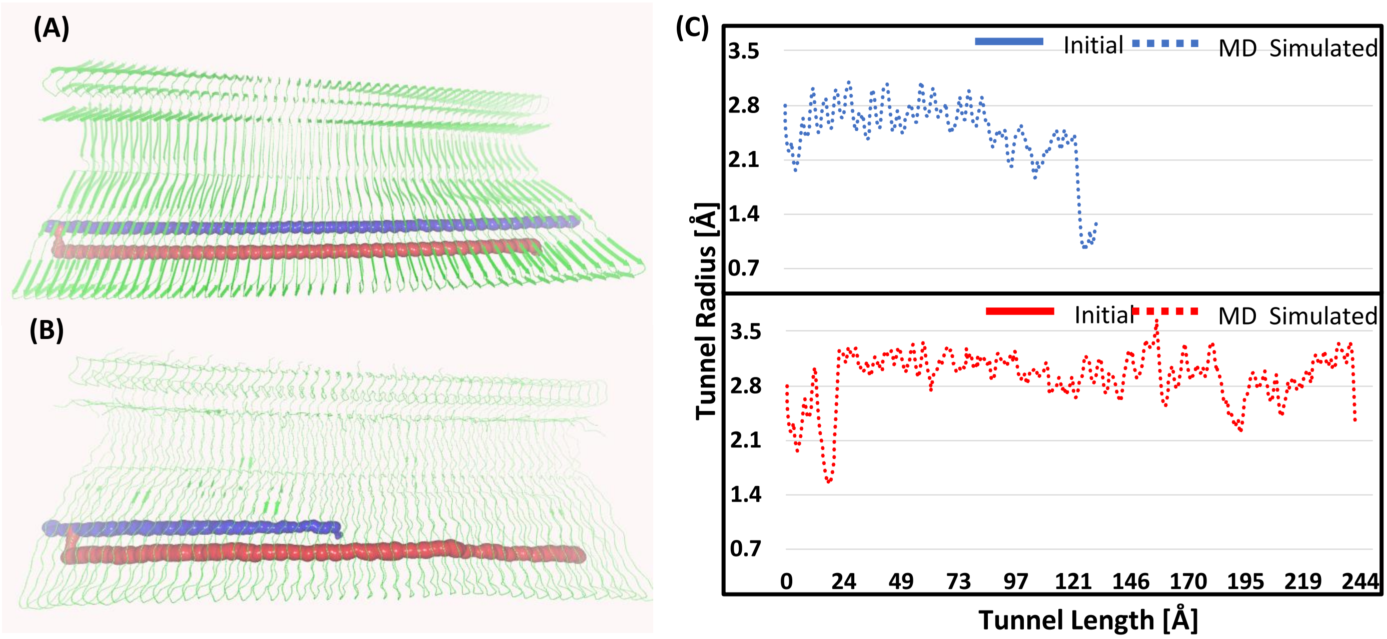

Supplementary Figure S4 Tunnels predicted in tau assembly modeled using cryo-EM filaments at the beginning (A) and after (B) MD simulation. Predicted tunnels (blue and red) and their radius in initial (continuous line) and MD simulated (dotted line) structure.

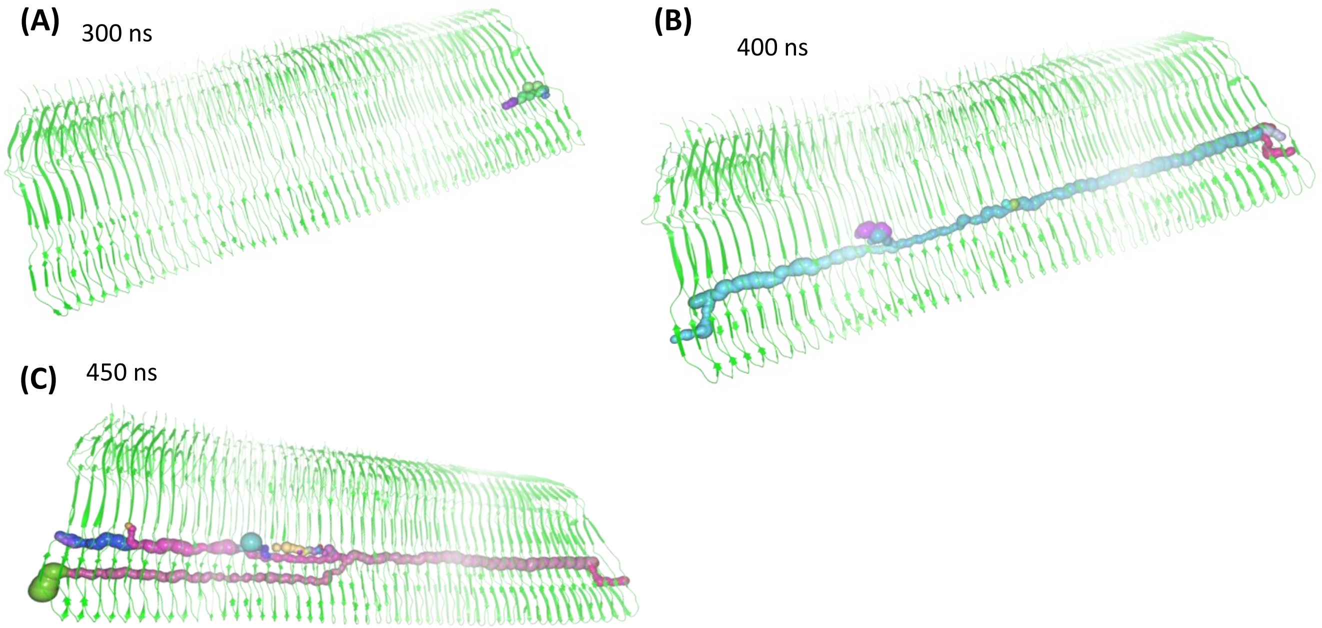

Supplementary Figure S5 Tunnels calculated in the tau structure from MD simulation after (A) 300 ns, (B) 400 ns and (C) 450 nanoseconds.
