# Supplementary material for: Pick’s Tau Fibril Shows Multiple Distinct PET Probe Binding Sites: Insights from Computational Modelling
Source: Int J Mol Sci. 2020 Dec 31;22(1):349. doi: 10.3390/ijms22010349 (PMC7796283; doi:10.3390/ijms22010349)
Supplement: Supplementary file 1 [file ijms-22-00349-s001.zip › Supplementary files/Table S1.docx]

**Table S1.** The non-bonded (∆E_ELE_ and ∆E_vdW_) and solvation energy term (∆E_GB_ and ΔE_SURF_) contribution to the MM/GBSA binding energy (ΔG_TOT_) of different PET probes in various binding sites. Binding energies (ΔG_TOT_) ± standard deviation of the energies over 1000 MD frames are shown in kcal·mol^-1^.

| AV-1451 |  | **ΔE_vdW_** | **ΔE_ELE_** | **ΔE_GB_** | **ΔE_SURF_** | **ΔG_TOT_** |
| --- | --- | --- | --- | --- | --- | --- |
|  | C1 | −35.5 ± 2.4 | −1.2 ± 7.3 | 13.8 ± 6.7 | −2.8 ± 0.2 | −25.8 ± 2.8 |
|  | S2 | −15.1 ± 1.8 | −1.5 ± 4.5 | 7.4 ± 4.4 | −1.5 ± 0.2 | −10.7 ± 1.6 |
|  | S5 | −25.8 ± 2.1 | −14.2 ± 3.1 | 22.1 ± 3.2 | −1.9 ± 0.1 | −19.9 ± 1.9 |
|  | S6 | −19.2 ± 8.1 | −8.5 ± 5.6 | 15.4 ± 6.8 | −1.6 ± 0.6 | −13.8 ± 6.9 |
|  | S7 | −32.6 ± 2.8 | −11.6 ± 4.2 | 21.2 ± 3.9 | −2.5 ± 0.2 | −25.5 ± 2.5 |
|  | S8 | −7.1 ± 6.9 | −5.8 ± 6.6 | 10.3 ± 7.6 | −0.7 ± 0.6 | −3.2 ± 5.0 |
| MK-6240 | C1 | −41.5 ± 3.5 | −13.5 ± 5.0 | 26.1 ± 3.6 | −3.2 ± 0.1 | −32.1 ± 4.1 |
|  | S4 | −7.5 ± 5.6 | −0.8 ± 5.7 | 5.0 ± 6.5 | −0.7 ± 0.5 | −3.9 ± 3.7 |
|  | S5 | −17.3 ± 3.6 | −13.7 ± 8.1 | 20.1 ± 8.0 | −1.5 ± 0.3 | −12.5 ± 3.3 |
|  | S9 | −11.6 ± 8.7 | −6.0 ± 6.5 | 11.6 ± 9.3 | −1.0 ± 0.7 | −7.1 ± 5.7 |
| PBB3 | C1 | −51.6 ± 2.5 | −10.6 ± 4.1 | 25.3 ± 3.4 | −4.0 ± 0.2 | −40.9 ± 2.7 |
|  | S1 | −37.1 ± 6.2 | −17.8 ± 4.7 | 32.1 ± 5.2 | −3.2 ± 0.5 | −26.0 ± 5.5 |
|  | S3 | −18.2 ± 1.7 | 2.4 ± 3.2 | 4.1 ± 3.2 | −2.0 ± 0.2 | −13.6 ± 1.6 |
|  | S4 | −25.5 ± 2.6 | −3.9 ± 3.4 | 14.9 ± 3.1 | −2.3 ± 0.2 | −16.9 ± 2.6 |
|  | S5 | −34.9 ± 3.5 | −16.7 ± 5.4 | 28.3 ± 4.8 | −2.9 ± 0.2 | −26.2 ± 3.3 |
|  | S6 | −35.6 ± 2.6 | −8.7 ± 4.6 | 23.1 ± 5.2 | −3.1 ± 0.2 | −24.3 ± 2.4 |
|  | S7 | −37.6 ± 2.6 | −12.7 ± 3.8 | 25.9 ± 3.5 | −3.3 ± 0.2 | −27.6 ± 2.6 |
|  | S8 | −38.4 ± 3.1 | −15.0 ± 3.9 | 28.8 ± 4.0 | −3.4 ± 0.2 | −27.9 ± 2.9 |
| PM-PBB3 | C1 | −63.3 ± 2.8 | −18.6 ± 7.0 | 36.8 ± 5.8 | −5.0 ± 0.2 | −50.0 ± 3.1 |
|  | S2 | −18.5 ± 5.8 | −10.7 ± 8.8 | 18.5 ± 9.8 | −2.0 ± 0.7 | −12.7 ± 4.6 |
|  | S3 | −15.9 ± 5.8 | −0.7 ± 4.5 | 7.2 ± 4.9 | −1.7 ± 0.7 | −11.2 ± 5.1 |
|  | S4 | −29.7 ± 3.0 | −1.0 ± 3.9 | 14.5 ± 3.2 | −2.6 ± 0.2 | −18.9 ± 2.4 |
|  | S8 | −46.4 ± 3.3 | −13.8 ± 7.0 | 29.7 ± 6.7 | −3.8 ± 0.3 | −34.2 ± 3.3 |
|  | S2n | −39.3 ± 2.8 | −13.4 ± 5.1 | 25.8 ± 4.5 | −3.3 ± 0.2 | −30.2 ± 3.2 |
| THK5351 | C1 | −45.2 ± 5.9 | −18.0 ± 5.0 | 33.6 ± 4.8 | −3.6 ± 0.3 | −33.2 ± 5.1 |
|  | S2 | −17.8 ± 1.9 | −1.3 ± 3.7 | 8.3 ± 3.7 | −1.9 ± 0.2 | −12.6 ± 1.8 |
|  | S5 | −18.3 ± 19.5 | −6.4 ± 5.8 | 14.8 ± 6.5 | −1.4 ± 0.4 | −11.3 ± 19.3 |
|  | S6 | −29.4 ± 3.1 | −12.4 ± 5.7 | 25.3 ± 4.6 | −2.8 ± 0.2 | −19.3 ± 2.9 |
|  | S7 | −32.6 ± 3.3 | −18.3 ± 6.9 | 31.1 ± 6.0 | −3.1 ± 0.2 | −22.8 ± 3.2 |
|  | S10 | −26.3 ± 11.3 | −14.7 ± 15.1 | 25.9 ± 17.7 | −2.3 ± 0.9 | −17.4 ± 8.7 |
| PiB | C1 | −36.2 ± 3.0 | −10.6 ± 3.2 | 22.9 ± 2.8 | −3.1 ± 0.2 | −27.0 ± 3.0 |
|  | S1 | −31.5 ± 2.7 | −8.2 ± 5.2 | 21.1 ± 5.3 | −2.7 ± 0.2 | −21.4 ± 2.9 |
|  | S2 | −16.5 ± 4.0 | −21.8 ± 4.5 | 26.8 ± 5.5 | −1.7 ± 0.3 | −13.1 ± 2.8 |
|  | S3 | −14.1 ± 2.0 | −4.2 ± 3.7 | 9.1 ± 3.6 | −1.5 ± 0.2 | −10.7 ± 1.7 |
|  | S4 | −8.8 ± 8.2 | −2.7 ± 5.2 | 6.8 ± 6.5 | −0.8 ± 0.7 | −5.5 ± 5.7 |
|  | S5 | −20.2 ± 3.8 | −4.7 ± 5.4 | 12.7 ± 4.9 | −1.6 ± 0.3 | −13.8 ± 3.4 |
|  | S6 | −20.0 ± 7.3 | −10.0 ± 9.8 | 18.2 ± 7.2 | −1.9 ± 0.5 | −13.7 ± 4.8 |
|  | S7 | −29.9 ± 2.2 | −18.5 ± 6.0 | 24.9 ± 4.7 | −2.4 ± 0.2 | −25.9 ± 2.6 |
|  | S9 | −15.9 ± 7.9 | −10.9 ± 9.7 | 17.6 ± 11.1 | −1.5 ± 0.7 | −10.7 ± 6.5 |
|  | S10 | −8.4 ± 8.8 | −4.5 ± 8.8 | 8.0 ± 9.6 | −0.7 ± 0.7 | −5.6 ± 6.9 |
|  | S11 | −23.0 ± 26.5 | −0.7 ± 3.3 | 8.6 ± 5.4 | −1.7 ± 0.8 | −16.8 ± 25.8 |
